# Case Report: Multimodal Imaging in a Rare Case of Morning Glory Disc Anomaly Complicated With Choroidal Ossification

**DOI:** 10.3389/fmed.2022.826860

**Published:** 2022-05-16

**Authors:** Shuya Wang, Xingrong Wang, Ying Wang, Hongsheng Bi

**Affiliations:** ^1^Department of Ophthalmology, Shandong University of Traditional Chinese Medicine, Jinan, China; ^2^Department of Ophthalmology, Affiliated Eye Hospital of Shandong University of Traditional Chinese Medicine, Jinan, China; ^3^Department of Ophthalmology Shandong Provincial Key Laboratory of Integrated Traditional Chinese and Western Medicine for Prevention and Therapy of Ocular Diseases, Jinan, China; ^4^Key Laboratory of Integrated Traditional Chinese and Western Medicine for Prevention and Therapy of Ocular Diseases in Universities of Shandong, Jinan, China; ^5^Eye Institute of Shandong University of Traditional Chinese Medicine, Jinan, China

**Keywords:** morning glory disk anomaly, choroidal osteoma, choroidal ossification, multimodal imaging, etiology analysis

## Abstract

**Purpose:**

The authors described a 7-year-old boy who was diagnosed with morning glory disc (MGD) anomaly in the right eye *via* fundus examination. However, during the head CT examination, a hyperdense choroidal lesion was discovered around the optic disc. Comprehensive investigations revealed that the lesion's characteristics were extremely consistent with choroidal osteoma (CO), so the patient was diagnosed with MGD with CO on his second visit. However, in the subsequent follow-up, the author discovered pigmentary alterations in the retinal pigment epithelium (RPE) in the patient's right eye. Finally, the diagnosis was corrected to MGD with choroidal ossification following a thorough etiological analysis. Meanwhile, the characteristics of choroidal ossification were described in detail through multimodal imaging in this article.

**Methods:**

Retrospective review of a case note.

**Conclusions:**

Similar to CO, choroidal ossification is the consequence of structured osseous tissue formation regulated by osteoblasts and osteoclasts. It consists of bone trabecular and vascular components and is difficult to be distinguished from CO on imaging examinations. In contrast to the congenital prevalence of CO, there are often incentives for the occurrence of choroidal ossification. These inducements will eventually mediate the inflammation in the eye, resulting in the activation of many cytokines and the production of choroidal ossification. Around one-third of patients with MGD will experience retinal detachment, and in certain cases, the subretinal fluid will be absorbed spontaneously, resulting in alterations to the RPE. These processes can activate inflammatory factors in the eye, bringing about a cascade of abnormalities, including the development of CO. Therefore, the proper diagnosis of disease should not be made exclusively on the basis of the imaging findings. A thorough analysis of the epidemiology and etiology is crucial.

## Introduction

Intraocular ossification is a kind of ectopic ossification which occurs when an extraskeletal bone tissue forms at the level of soft, well-vascularized tissues (1). Choroidal ossification, as the name implies, is the acquired ectopic ossification that happens in the choroid and is regulated by osteoblasts and osteoclasts. An etiologic factor for intraocular heterotopic bone formation could be chronic inflammation, trauma, absolute glaucoma, or a long-standing retinal detachment. Chronic ocular inflammation, bone morphogenetic proteins, and mesenchymal stem cells have all been implicated in the process of intraocular ossification, according to recent research.

Morning glory disc (MGD) anomaly was first described by Kindler in 1970 (2). It is a congenital optic disc anomaly of unknown etiology characterized by optic disc enlargement, conical excavation, peripapillary pigmentary disturbance, the central tuft of glial tissue, and an increased number of straight retinal vessels radially emerging from the disc margin. Retinal detachment was reported to appear in approximately one-third of patients with MGD, and a spontaneous absorption of subretinal fluid has been observed in some of these cases, leaving pigmentary alterations in the retinal pigment epithelium (RPE).

We report a case of MGD with choroidal ossification and describe the morphology of choroidal ossification using a multimodal image system.

## Case Report

**Figure 1:**
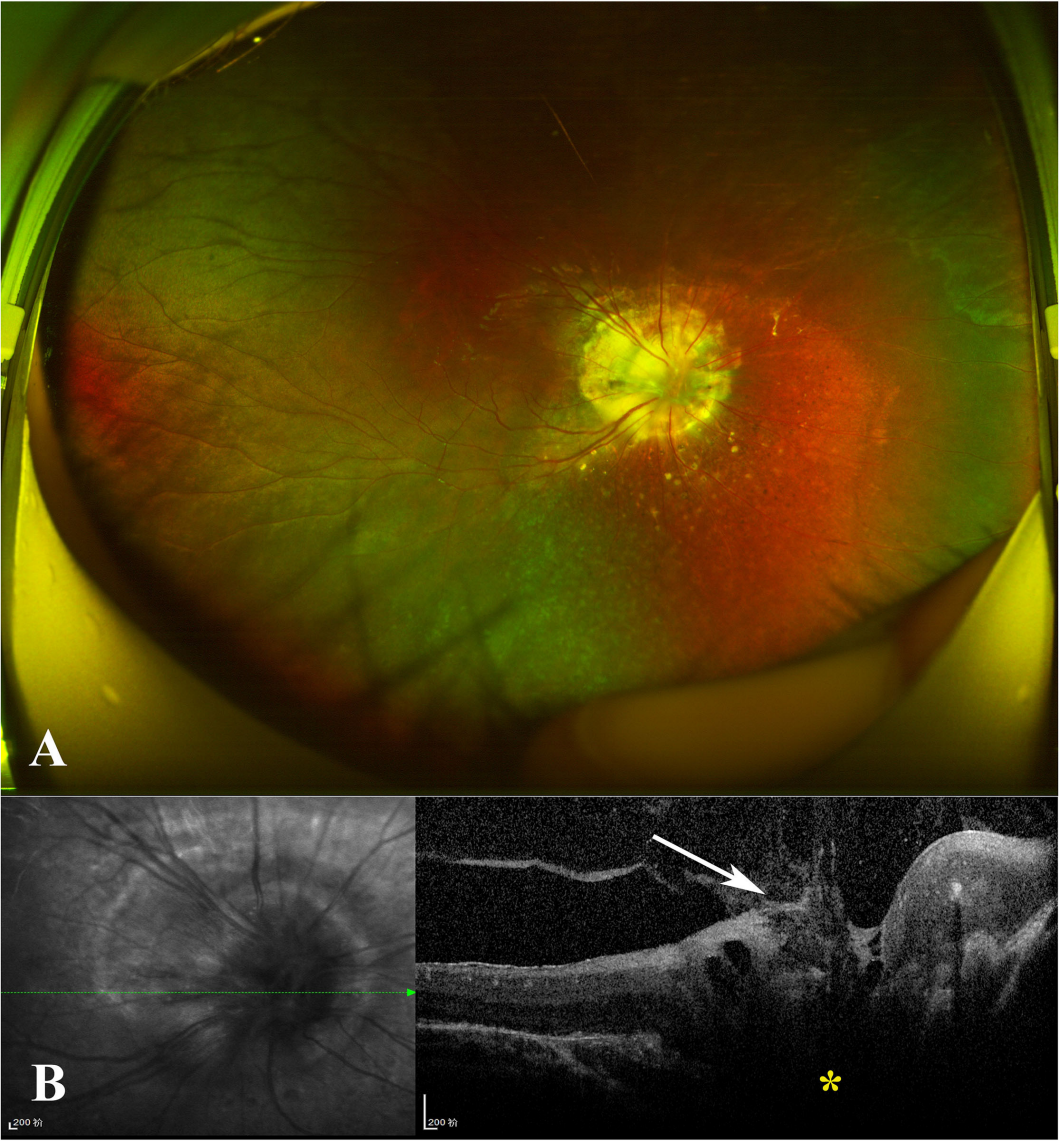
**(A)** Widefield color fundus photograph of the right eye revealed a large optic disc centrally covered with glial tissue and encircled with chorioretinal pigmentary disturbance, as well as straight retinal vessels radially issuing from the disc margin with increased numbers. **(B)** Spectral-domain optical coherence tomography (SD-OCT) revealed the conical excavation of the optic disc (yellow star) with a central tuft of glial tissue on top (white arrow).

In November 2019, a 7-year-old boy was referred to our ophthalmology department due to poor vision in his right eye after a pre-school physical assessment. The best-corrected visual acuity (BCVA) was 20/160 in the right eye with an objective refraction of +1.00 diopter and 20/20 in the left. There was no evident strabismus in either eye. The anterior segment, intraocular pressures (IOPs), and ocular adnexa of both eyes were normal. A widefield color fundus image of the right eye revealed a large optic disc covered with glial tissue in the center and bordered by chorioretinal pigmentary disturbance, as well as straight retinal vessels extending radially from the disc margin in increased numbers (Figure 1A). In the left eye, the fundus appeared normal. The spectral-domain optical coherence tomography (SD-OCT) showed a conical excavation of the optic disc with a central tuft of the glial tissue on top (Figure 1B). His parents denied any histories of familial disease, abnormalities in pregnancy, prematurity, or low birth weight. For the characteristic fundus manifestations, the patient was preliminarily diagnosed with MGD anomaly and subjected to other systemic examinations, which were normal.

**Figure 2:**
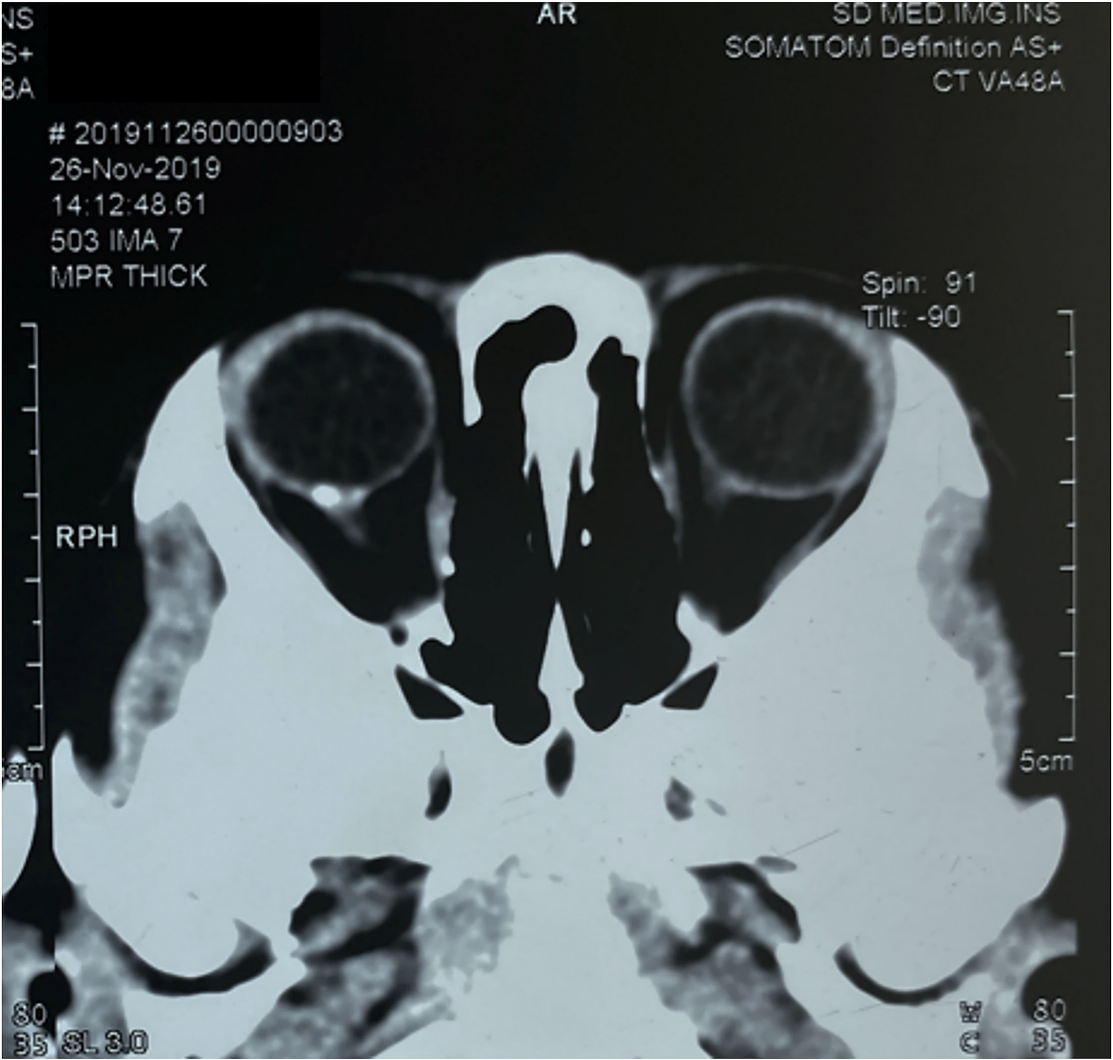
Computerized tomography scan of the orbit demonstrated a hyperdense choroidal plaque with the same density as bone at the level of the optic disc in the right eye.

**Figure 3:**
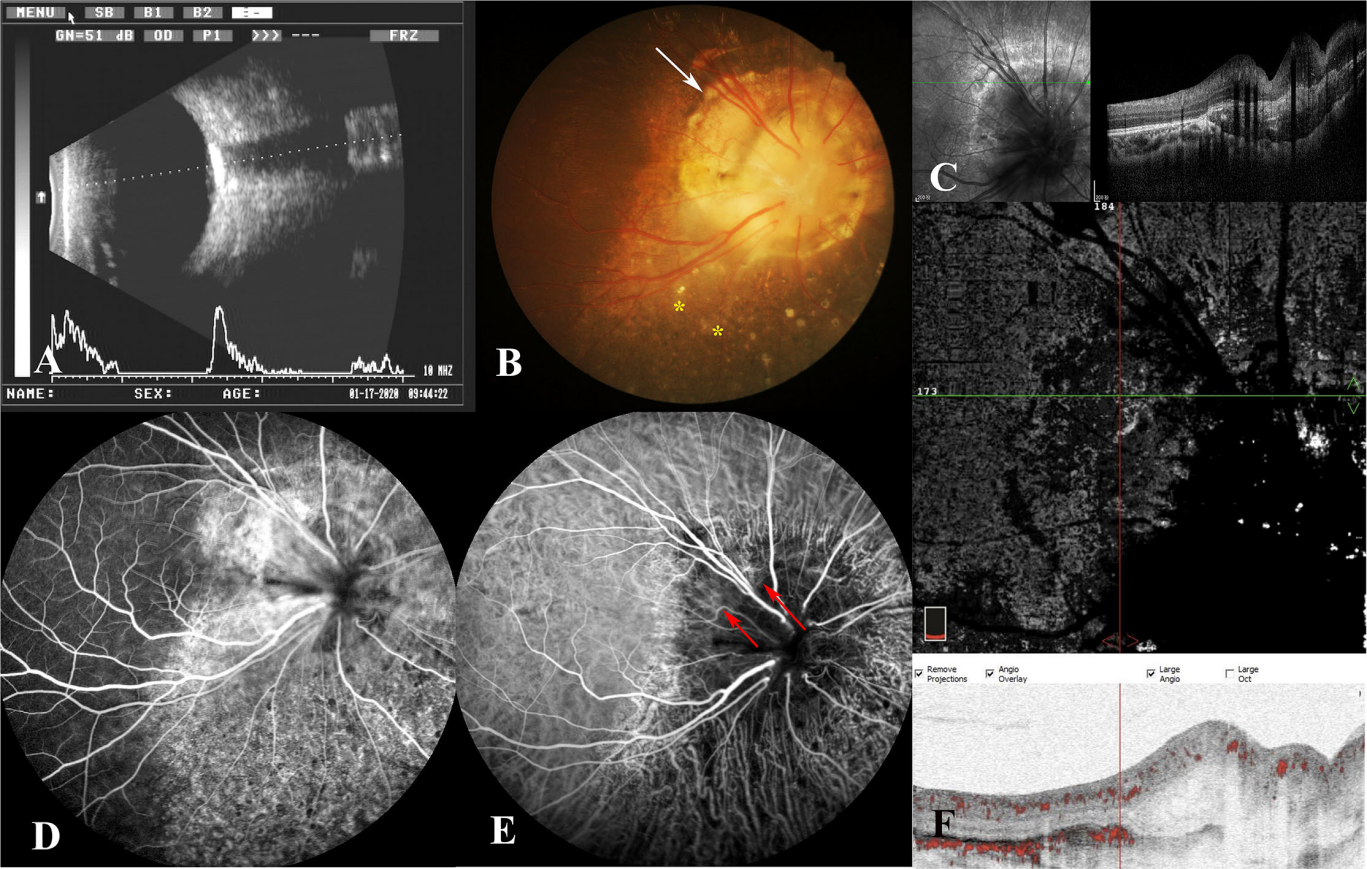
**(A)** B-scan ultrasonography demonstrated a highly reflected mass covering the optic disc, and a high echo spike originating from the surface of the lesion was detected on the combined A-scan. **(B)** Fundus photography showed a yellowish-white lesion around the optic disc (white arrow) and mottling of the retinal pigment epithelium (RPE) (yellow stars). **(C)** On enhanced depth imaging OCT (EDI-OCT), a well-defined peripapillary choroidal lesion with hyperreflective lamellar lines in the inner part and spongiform pattern in the outer part were depicted. **(D)** The late phase of FA revealed diffuse late staining around the optic disc and punctate hypofluorescence inferiorly. **(E)** The indocyanine green angiography (ICGA) illustrated early hypofluorescence and diffuse late staining in the juxtapapillary area. In the early phase, the patterns of vessels were obtained supranasally to the optic disc (red arrow). **(F)** The blood flow signals were demonstrated among the lesion on OCT angiography (OCTA).

Magnetic resonance imaging (MRI) and CT scan were also performed, and intracranial abnormalities were ruled out. However, the CT scan of the orbit demonstrated a hyperdense choroidal plaque with the same density as bone at the level of the optic disc in the right eye (Figure 2). Moreover, further ocular examinations were performed. The b-scan ultrasonography revealed a highly reflected mass covering the optic disc detected at even a very low gain, whereas the vector scan showed a high echo spike originating from the surface of the lesion (Figure 3A). On dilated funduscopic examination, a yellowish-white and slightly elevated lesion around the optic disc was detected, which had previously been considered a common annulus of chorioretinal pigmentary changes in MGD (Figure 3B). The enhanced depth imaging (EDI-OCT) image depicted a well-defined peripapillary choroidal lesion with hyperreflective lamellar lines in the inner part and spongiform pattern in the outer part, which replaced the normal choriocapillaris (Figure 3C). The fluorescein angiography (FA) showed early patchy hyperfluorescence and diffuse late staining around the optic disc (Figure 3D). On indocyanine green angiography (ICGA), there was an area of early hypofluorescence and diffuse late staining corresponding to the elevated lesion. It was noteworthy that during the early stages of ICGA, supranasal to the optic disc, the vascular patterns were obtained (Figure 3E), and the 6 × 6 mm OCT angiography (OCTA) centered at these locations delineated clusters of linear vessels on *en face* visualization. Additionally, a cross-sectional OCTA b-scan indicated blood flow signals in these lamellar choroidal lesions (Figure 3F). Based on the exhaustive examinations described above, the diagnosis of MGD with choroidal osteoma (CO) was made for this patient on his second visit in January, 2020.

At the third follow-up 3 months later, there were no major changes in the right eye, except that some details that had previously been neglected were observed this time. The mottling of the RPE around the optic disc was detected on the fundus imaging of the right eye (Figure 3A). Additionally, by reviewing the FA pictures of the right eye, the punctate hypofluorescence in the inferior region of the optic disc was discovered (Figure 3D). These changes are uncommon in CO, whereas they have been reported in MGD with retinal detachment. The details of these new findings compelled us to reassess the validity of the diagnosis of osteoma in this patient. Although the examination results indicated that the peripapillary lesion in this patient was highly consistent with CO, CO is more common in young women, and as the disease progresses, dynamic changes such as decalcification will occur. The stability of the peripapillary lesion in the patient's right eye, together with the RPE disruption surrounding the optic disc, suggested another alternative, choroidal ossification. For this patient, we arrived at the ultimate diagnosis of MGD with choroidal ossification, and the correction of the refractive errors was made for his right eye. The patient has been constantly monitored, and the patient's right eye remained stable at the subsequent follow-up.

## Discussion

Unlike optic nerve coloboma, MGD is almost universally a sporadic condition (3). Most cases of MGD, like the case reported here, are isolated and unassociated with systemic anomalies. Nevertheless, the reported associations are not rare including agenesis of the corpus callosum, basal encephalocele, congenital forebrain abnormalities, persisting embryonal infundibular recess (PEIR), etc. Therefore, evaluating the cerebral architecture and related anomalies is critical. Although the patient completed a comprehensive examination and no concomitant systemic abnormalities were discovered, a CT scan revealed a peripapillary lesion of bone density at the choroid level. Then, the bony features of the lesion were clarified by ultrasound, FA, OCT, and other more comprehensive examinations. Especially on EDI-OCT, the lesion exhibited horizontal hyperreflective lamellar lines corresponding to the bone lamella, hyperreflective horizontal denser lines (cement line), horizontal and vertical tubules correlating with vascular channels, and a speckled appearance resembling the spongy trabecular or compact bone. The osseous characteristics of the lesion and the highly consistent imaging manifestations with CO prompted us to conclude the diagnosis of CO in the first place (4, 5).

Choroidal osteoma is an ossifying tumor that affects the choroid with unknown etiology; its natural progression may involve tumor development, calcification, and decalcification (6). It usually occurs unilaterally with a predilection for women at a relatively young age (5–7). The combination of other congenital ocular anomalies is rarely seen in reports of CO (8). RPE alterations in the periphery of the lesion have rarely been seen in reported cases. The patient, in this case, was a male child who had MGD as well as a peripapillary osseous lesion in his right eye. The overlying RPE and outer retina of the lesion were normal, but the RPE around the lesion revealed a mottling appearance. These clinical findings led us to question the accuracy of the diagnosis of CO. Another secondary lesion with bony features, choroidal ossification, is a more likely combination or secondary lesion than CO in this MGD case.

Distinct from calcification, choroidal ossification, manifesting itself as structured bone tissue regulated by osteoblasts and osteoclasts, is a recognized entity with a more complicated mechanism of formation. It immensely resembles to CO in terms of morphological and imaging features. Choroidal ossification commonly has a chronic antecedent including retinal detachment, trauma, chronic ocular inflammation, absolute glaucoma, etc. In general, the ossification requires osteogenic precursor cells that induce ossification, and in the eye, the ossification results from osteoblastic transformation of the RPE. RPE is now known to be a pluripotent cell with the ability to differentiate into mesenchymal phenotypes, including fibroblasts and bone (9). Chronic eye diseases including phthisis, trauma, glaucoma, and retinal detachment are often associated with intraocular inflammation, and inflammatory cells release interleukin-1 (IL-1) or tumor necrosis factor alpha (TNF-α), which stimulates the RPE to produce transforming growth factor beta1 (TGF-β1) and bone morphogenetic proteins 7 (BMP-7). TGF β1 triggers the transformation of the mesenchymal epithelium into fibrous metaplasia of the RPE cells. BMP-7 can counteract the effect of TGF-β1 and thus hinder this transformation process. But at the same time, BMP-7 promotes transforming process of the degenerating RPE to osteoblasts (10). This explains why ossification tends to occur in the subretinal area. Besides, ossification requires a rich vascular supply; therefore, the peripapillary region is always involved. The few preretinal ossification occurs after the migration of RPE cells from the subretinal space to the preretinal surface or vitreous cavity (through retinal breaks) (11). Moreover, as a probable source of pluripotent stem cells, pericytes were found to be capable of differentiation into osteoprogenitor cells and meanwhile generating mineralized bone matrix (12, 13).

In conclusion, proliferation and transdifferentiation of the RPE cells appear to be prerequisites for intraocular ossification. Back to our case, as an ocular congenital abnormality, MGD cannot be the antecedent for choroidal ossification. Like most patients with MGD, the patient in this case had poor vision since childhood, so he was unable to adequately perceive or describe changes in visual acuity. Thus, we should be attentive to the ocular history or changes in the state of the inherent disease that may be overlooked by the patient. MGD with combined retinal detachment has never been an accidental event. Several pathogenic mechanisms have been put forward: indiscoverable retina breaks (14), communication between the subretinal and subarachnoid spaces (15), fluid leakage from abnormal intra-optic disc vessels or peripapillary choroid, etc. (16). Additionally, the spontaneous resolution of the retinal detachment has been observed, which was speculated by the pigmentary changes in RPE (17). Netan et al. also reported a case of MGD with nonrhegmatogenic retinal detachment in which the subretinal fluid is absorbed spontaneously, leaving RPE mottling (18). Here in this case, through a careful reading of the fundus photography and fundus FA (FFA), the changes of RPE were also discovered, which highly suggested the previous retinal detachment and spontaneous resolution of the subretinal fluid. The detachment and spontaneous reattachment of the retina triggers the inflammation of the eye, which then sets off the subsequent multilineage differentiation of the RPE cells and the onset of ossification. Additionally, choroid ossification in this case might also be a secondary process following a (birth-)trauma. To summarize, the difference between secondary choroidal ossification formation and primary CO can be explained by the different intensities of osteogenic (RPE) stimulation. So, for our case, while making the diagnosis of MGD, the diagnosis of concomitant Choroidal Ossification is more comprehensive than CO. Unlike CO, which is characterized by persistent growth, decalcification, or neovascularization within the tumor, choroidal ossification is more stable. We do not need to give those treatment recommendations for CO, such as transpupillary thermotherapy (TTT), photodynamic therapy (PDT), or anti-vascular endothelial growth factor (anti-VEGF) therapy, to this patient. However, close monitoring is required.

To our knowledge, this case is the first report of MGD associated with choroidal ossification. Furthermore, the characteristics of choroidal ossification were thoroughly detailed here through comprehensive imaging evaluations.

## Data Availability Statement

The raw data supporting the conclusions of this article will be made available by the authors, without undue reservation.

## Ethics Statement

Written informed consent was obtained from the individual(s), and minor(s)' legal guardian/next of kin, for the publication of any potentially identifiable images or data included in this article.

## Author Contributions

SW is the first collector of case and is responsible for the drafting of the article. XW and YW participated in the reading of examination reports. HB provided assistance for the writing of the article. All authors contributed to the article and approved the submitted version.

## Funding

This study was supported by Key research and development program of Shandong Province (item number: F2021LCZX09).

## Conflict of Interest

The authors declare that the research was conducted in the absence of any commercial or financial relationships that could be construed as a potential conflict of interest.

## Publisher's Note

All claims expressed in this article are solely those of the authors and do not necessarily represent those of their affiliated organizations, or those of the publisher, the editors and the reviewers. Any product that may be evaluated in this article, or claim that may be made by its manufacturer, is not guaranteed or endorsed by the publisher.
